# Analysis on the risk factors for organ damage in patients with systemic lupus erythematosus: a cross-sectional single-center experience

**DOI:** 10.1590/1516-3180.2018.0258060219

**Published:** 2019-07-15

**Authors:** Valentina Živković, Branka Mitić, Bojana Stamenković, Sonja Stojanović, Biljana Radovanović Dinić, Miodrag Stojanović, Vladimir Jurišić

**Affiliations:** I MD, PhD. Assistant Professor, Faculty of Medicine, University of Niš, and Institute for Treatment and Rehabilitation “Niška Banja”, Niš, Serbia.; II MD, PhD. Assistant Professor, Faculty of Medicine, University of Niš, and Clinic of Nephrology, Clinical Centre, Niš, Serbia.; III MD, PhD. Assistant Professor, Faculty of Medicine, University of Niš, andInstitute for Treatment and Rehabilitation “Niška Banja”, Niš, Serbia.; IV MD, PhD. Assistant Professor, Faculty of Medicine, University of Niš, and Institute for Treatment and Rehabilitation “Niška Banja”, Niš, Serbia.; V MD, PhD. Associate Professor,Faculty of Medicine, University of Niš, and Clinic for Gastroenterology and Hepatology, Clinical Centre, Niš, Serbia.; VI MD, PhD. Associate Professor, Faculty of Medicine, University of Niš, and Public Health Institute, Niš, Serbia; VII MD, PhD. Professor,Faculty of Medical Sciences, University of Kragujevac, Kragujevac, Serbia.

**Keywords:** Lupus erythematosus, systemic, Risk factors, Quality of life

## Abstract

**BACKGROUND:**

Organ damage in patients with systemic lupus erythematosus (SLE) occurs as a consequence of the disease itself, the therapy applied and the accompanying conditions and complications. Organ damage predicts further organ damage and is associated with an increased risk of death.

**OBJECTIVE:**

This study aimed to assess the degree of irreversible organ changes in SLE patients, using the Systemic Lupus International Collaborating Clinics/American College of Rheumatology (SLICC/ACR) damage index (SDI); to establish correlations between organ damage and disease activity, quality of life, intensity of fatigue and serological factors; and to ascertain the risk factors for organ damage.

**DESIGN AND SETTING:**

Cross-sectional single-center study conducted at the Institute for Treatment and Rehabilitation “Niška Banja”, Niš, Serbia.

**METHODS:**

83 patients with SLE were enrolled: 58 patients formed the group with organ damage (SDI ≥ 1), and 25 patients without organ damage served as controls (SDI = 0).

**RESULTS:**

Organ damage correlated with age (P = 0.002), disease duration (P = 0.015), disease activity (grade 1, P = 0.014; and grade 2, P = 0.007), poor quality of life, severe fatigue (P = 0.047) and treatment with azathioprine (P = 0.037). The following factors were protective: use of hydroxychloroquine (P = 0.048) and higher scores obtained for the physical (P = 0.011), mental (P = 0.022) and general health (P = 0.008) domains.

**CONCLUSION:**

It is very important to evaluate risk factors for organ damage in the body, including physicians’ overall assessment, to try to positively influence better treatment outcomes.

## INTRODUCTION

The multisystemic nature of systemic lupus erythematosus (SLE), its involvement of vital organs and its unpredictable disease course with exacerbations and remissions give rise to the possibility of development of irreversible changes in individual organs even at early phases of the disease, and particularly after several years.^1^ Tissue and organ damage occurs as a consequence of the disease itself, the therapy applied (primarily corticosteroid and cytostatic), and the accompanying conditions and complications. Tissue and organ damage are associated with an increased risk of death.^2^^,^^3^

Adequate evaluation of disease activity, assessment of organ damage using the Systemic Lupus International Collaborating Clinics/American College of Rheumatology (SLICC/ACR) damage index for SLE(SDI)^4^ and quality of life assessment among SLE patients contribute towards better surveillance and treatment, and improved prognosis for the disease.^5^^,^^6^

## OBJECTIVE

Our aims in this paper were to examine the degree of irreversible organ changes in SLE patients using the SDI; to establish correlations between organ damage and disease activity, biological disease markers, quality of life and severity of fatigue; and to ascertain the risk factors for organ damage in SLE patients.

## METHODS

Before enrollment in the study, all the examinees were informed about the study objectives and their informed written consent was obtained. The study was approved by our institution’s ethics committee (date: November 28, 2013;number: 03-14421/1).

All patients with SLE who were hospitalised in the Rheumatology Clinic, Institute “Niška Banja” during 2012/13, with the duration of the disease longer than six months, were eligible for this study. Thepatients’ diagnosis of SLE was made on the basis of the revised ACR criteria of 1997.^7^ This cross-sectional study involved 83 patients with SLE (77 women and 6 men), with mean age of 45.8 ± 9.2 years and average disease duration of 10.6 ± 7.9 years.

Disease activity was assessed using the Systemic Lupus Erythematosus Disease Activity Index (SLEDAI) and the physician’s global assessment.^8^ SLEDAI assesses activity in nine organ systems based on the presence or absence of 24 variables at the time of the examination and up to 10 days after the examination. The values range from 0 to 105. Physician’s global assessment grade was assigned by the researchers on the basis of anamnesis, physical examination and supplementary investigations. The grade ranged from 0 to 3 (0 - no activity; 1 - mild activity; 2 - moderate activity; 3 - severe disease).

The degree of organ damage was evaluated using the standardized damage index (SDI).^4^ The SDI assesses damage in nine organ systems and three disease complications. It measures the presence of irreversible changes in the eyes, skin and neuropsychiatric, renal, pulmonary, cardiovascular, peripheral vascular, gastrointestinal and musculoskeletal systems, along with the presence of gonadal insufficiency, diabetes mellitus and malignancy. Each of the characteristics adds an appropriate number of points to the overall score and is precisely defined in the SDI glossary. The total possible score for the damage index is 47.

Based on the damage index value, the SLE patients were divided into two groups: a study group comprising 58 patients (69.9%) with organ damage (SDI ≥ 1); and a control group comprising 25patients (30.1%) without organ damage (SDI = 0). At the disease onset, the two groups were homogenous regarding the factors of gender and age. Quality of life was assessed on the basis of the standardized Medical Outcome Survey Short Form 36 (SF-36).^5^ In this study, the values of three summary domains were primarily considered: physical (SF-36P), mental (SF-36M) and general health (SF-36G). The SF-36 questionnaire contains 36 questions in total, grouped into eight domains, along with a question relating to status change. The responses are scored from 0 to 100, in accordance with the key that is made available, such that higher scores indicate better quality of life.

The severity of fatigue was assessed using the Fatigue Severity Scale(FSS).^9^ The FSS consists of nine statements for which possible answers graded from 1 to 7 are presented. The questions in the FSS relate to the preceding 14 days. Fatigue is graded as serious if the average value on the Fatigue Severity Scale exceeds the grade of 4.

The following demographic factors were observed: gender, age, disease duration, age at disease onset, time elapsed until diagnosis and education level (0-12 years or over 12 years). The following clinical factors were observed: number of diagnostic criteria, SLEDAI, physician’s global assessment, hypertension, proteinuria, antiphospholipid syndrome, osteopenia, osteoporosis, cardiovascular diseases, involvement of the kidneys and central nervous system (CNS), severity of fatigue and quality of life expressed in terms of the physical, mental and general health domains. Thefollowing serological factors were determined: presence of anti-double-stranded deoxyribonucleic acid (anti-dsDNA), antinucleosome and anti-C1q antibodies; and the levels of monocyte chemoattractant protein-1 (MCP-1) in the serum (sMCP-1) and urine (uMCP-1). The presence of antibodies was determined using the enzyme-linked immunosorbent assay (ELISA) test in an Alegria automated ELISA reader*,* manufactured by Orgentec(Germany). The levels of sMCP-1 and uMCP-1 were measured using the sandwich enzyme immunosorbent assaymethod, in accordance with the manufacturer’s instructions (R&D Systems, Inc.,Minneapolis*,* USA). The therapeutic factors used included prednisone, hydroxychloroquine, azathioprine, mycophenolate mofetil and pulse doses of cyclophosphamide.

Statistical calculations were performed using the Statistical Package for the Social Sciences (SPSS) software, version 20. Thedescriptive statistical analysis involved the following statistical parameters: arithmetic mean, standard deviation, range (minimum-maximum), absolute frequency (n) and structure index (%). Using the analytical statistical methodology, the statistical significance of the differences in frequency of appearance of certain characteristics in all examinees and according to group was measured.

The statistical testing was done using the chi-square test, or Fisher’s test for frequencies below five units. Comparisons of the mean values of characteristics between the groups were made using the t test for independent samples. Pearson’s correlation for parametric samples was used to measure the associations of particular characteristics, through correlation analysis. The predictive impact of individual variables on the dependent outcome variable was assessed using univariate and multivariate regression analyses. All the factors that were significant in the univariate model were included in a logistic multivariate analysis (“enter” method). An evaluation error level of below 5% (P < 0.05) was used at the threshold of statistical significance.

## RESULTS

The parametric characteristics of the examinees in the study and control group are shown in Table 1. The patients in the study group with organ damage and SDI ≥ 1 were statistically significantlyolder than those in the control group (47.9 ± 8.7 versus 40.8 ± 8.5 years; t = 3.464; P = 0.001)and presented longer disease duration (12.1 ± 8.6 versus 7.3 ± 4.8 years; t = 2.617; P=0.011). Wedid not find any statistically significant difference in the age at disease onset between the groups studied (t = 1.056; P = 0.294), or any significant difference in time elapsed from the onset of symptoms todiagnosis (t = 0.725; P = 0.471).

**Table 1.** Participants’ characteristics

Variables	Group with organ damage		Control group		t	P
	mean	SD	mean	SD		
Age (years)	47.9	8.7	40.8	8.5	3.464	0.001
Disease duration (years)	12.1	8.6	7.3	4.8	2.617	0.011
Age at disease onset (years)	35.9	9.9	33.5	7.9	1.056	0.294
Time elapsed until diagnosis (months)	14.2	16.5	11.5	12.5	0.725	0.471
Age at diagnosis (years)	37.0	10.1	34.3	8.1	1.155	0.252
SLEDAI	11.8	6.6	9.0	7.8	1.655	0.102
Physician’s global assessment	1.5	0.8	1.1	1.0	1.824	0.072
SF-36 physical health score	30.6	24.5	40.8	25.6	2.773	0.008
SF-36 mental health score	43.7	25.8	58.5	25.5	2.417	0.018
SF-36 global health score	35.0	22.8	51.2	24.5	2.895	0.005
Fatigue (FSS)	6.0	1.4	5.4	1.6	1.889	0.062

SD = standard deviation; SLEDAI = systemic lupus erythematosus disease activity index; SF-36 = medical outcome survey short form 36; FSS = fatigue severity scale.

The mean values of the activity index, SF-36 survey scores and fatigue scale are also presented in Table 1. There was no significant difference between the groups studied regarding disease activity and severity of fatigue, but the group with organ damage had significantly lower quality of life than the controls.

The mean value of the damage index for all the SLE patients was 1.8 ± 2.0 (median 1, minimum 0 and maximum 9); 25 patients (30.1%) did not have any organ damage (SDI = 0), 21 patients (25.3%) presented SDI = 1; 20 patients (24.1%) had SDI = 2 or 3; and 17 patients (20.5%) had SDI ≥ 4. Regarding specific organ damage, neuropsychiatric and musculoskeletal changes were most common, found in 23patients (27.7%). In 21 patients (25.3%), cardiovascular changes were seen, while eye lesions were found in 14 patients (16.9%). Renaland pulmonary changes were seen in 13 patients (15.7%), skin lesions in three cases (3.6%) and gastrointestinal changes were observed in two patients (2.4%). Malignancies were encountered in five patients (6.0%), and diabetes mellitus in two cases (2.4%).

There were statistically significant positive correlations between SDI and age (r = 0.348; P = 0.001), disease duration (r= 0.412; P<0.001), SLEDAI (r = 0.359; P = 0.001), physician’s global assessment (r = 0.357; P = 0.001) and fatigue (r =0.296; P =0.007). Negative correlations with quality of life were established in relation to SF-36P (r = -0.389; P < 0.001), SF-36M (r=-0.314; P = 0.004) and SF-36G (r = -0.386; P < 0.001), meaning that more extensive organ damage was associated with poorer quality of life. SDI did not show correlations with the levels of anti-dsDNA, antinucleosome or anti-C1q antibodies, or with the levels of sMCP-1 and uMCP-1. SLEDAI scores correlated positively with the following study parameters: anti-dsDNA antibodies (r=0.228; P<0.05), antinucleosome (r = 0.396; P < 0.001), anti-C1q antibodies (r=0.260; P < 0.05), sMCP-1 (r = 0.318; P<0.01) and uMCP-1 (r = 0.431; P < 0.001).

Table 2shows the results from univariate regression analysis on the independent demographic, serological, clinical and therapeutic parameters in the groups studied with and without organ damage. Among the demographic factors, the statistically significant independent factors favoring organ damage were the age of the examinees viewed as a continuous value (odds ratio, OR = 1.094; P = 0.002) and disease duration expressed as a continuous value (OR = 1.096; P = 0.015). Disease duration of over 10 years was recognized as a statistically significant risk factor, with a risk that was up to three times higher than that of disease duration of less than five years (OR = 3.368; P = 0.045). Among the clinical risk factors, disease activity (expressed as physician’s global assessment grade) and severe fatigue were considered significant. Regarding physician’s global assessment, grade 1 was a risk factor six times higher than grade 0 (OR = 5.800; P = 0.014) and grade 2 (OR = 10.667; P = 0.007) was a risk factor ten times higher, and these results were considered statistically significant. The patients with scores over 4 on the fatigue scale were exposed to a risk of organ damage that was three times higher than those with values ≤ 4 on this scale (OR = 3.370; P = 0.047). Higher values for the physical (OR = 0.976; P = 0.011), mental (OR = 0.978; P = 0.022) and general health (OR = 0.972; P = 0.008) domain scores were statistically significant protection factors against organ damage. Hydroxychloroquine use was a protection factor against organ damage (OR = 0.378; P = 0.048), while azathioprine treatment was a risk factor (OR = 9.143; P = 0.037). Serological factors, including the levels of anti-dsDNA, antinucleosome and anti-C1q antibodies, along with sMCP-1 and uMCP-1, were not found to be risk factors for organ damage.

**Table 2.** Univariate regression analysis on parameters evaluated among systemic lupus erythematosus patients

Factor		SDI = 0	SDI ≥ 1	OR	95% CI	P
		n (%)	n (%)			
Gender	[m]	0 (0.0)	6 (10.3)			
	f	25 (100.0)	52 (89.7)	0.000	0.000	0.999
Age				1.094	1.032-1.160	0.002
Age at disease onset				1.028	0.997-1.082	0.292
Disease duration				1.096	1.018-1.180	0.015
Time elapsed until diagnosis				1.013	0.978-1.048	0.469
Number of criteria at diagnosis				0.757	0.515-1.114	0.158
SLEDAI				1.065	0.978-1.149	0.106
Anti-dsDNA Ab				0.995	0.989-1.001	0.122
Positive anti-dsDNA Ab		19 (32.8)	39 (67.2)	0.648	0.223-1.888	0.427
Antinucleosome Ab				0.994	0.998-1.000	0.066
Positive antinucleosome Ab		20 (32.3)	42 (27.6)	0.656	0.211-2.045	0.468
Anti-C1q Ab				0.983	0.936-1.004	0.112
Positive anti-C1q Ab		11 (42.3)	15 (57.7)	0.444	0.166-1.188	0.106
Co-positivity for three antibodies		9 (39.1)	14 (60.9)	0.566	0.205-1.560	0.271
sMCP1				1.000	0.999-1.000	0.179
uMCP1				0.996	0.991-1.000	0.074
Prednisone dose				1.023	0.971-1.077	0.399
Hydroxychloroquine		15 (41.7)	21 (58.3)	0.378	0.144-0.991	0.048
Azathioprine		1 (5.9)	16 (94.1)	9.143	1.140-73.301	0.037
Hydroxychloroquine + Azathioprine		4 (28.6)	10 (71.4)	1.094	0.308-3.886	0.890
Cyclophosphamide pulsed dose		6 (31.6)	13 (77.4)	0.915	0.303-2.765	0.875
Mycophenolate mofetil		4 (33.3)	8 (66.7)	1.190	0.323-4.386	0.793
Physician’s global assessment	[0]	8 (66.7)	4 (33.3)			
	1	10 (25.6)	29 (74.4)	5.800	1.432-23.496	0.014
	2	3 (15.8)	16 (84.2)	10.667	1.909-59.615	0.007
	3	4 (30.8)	9 (69.2)	4.500	0.837-24.183	0.080
Antiphospholipid syndrome		1 (7.7)	12 (92.3)	6.261	0.768-51.068	0.087
Education (years)	[≤ 12]	21 (30.0)	49 (70.0)			
	> 12	4 (30.8)	9 (69.2)	0.964	0.267-3.982	0.956
Hypertension		13 (31.0)	29 (69.0)	0.923	0.361-2.359	0.867
Proteinuria > 0.5 g		8 (28.6)	20 (71.4)	1.118	0.412-3.039	0.826
Osteopenia and osteoporosis	[normal BMD]	7 (33.3)	14 (66.7)			
	osteopenia	17 (32.7)	35 (67.3)	1.029	0.351-3.021	0.958
	osteoporosis	1 (10.0)	9 (90.0)	4.500	0.471-42.970	0.191
Lupus nephritis		2 (28.6)	5 (71.4)	0.882	0.134-5.815	0.896
Lupus CNS		2 (12.5)	14 (87.5)	3.659	0.765-17.501	0.104
Coronary disease, stroke and peripheral atherosclerosis		0 (0.0)	7 (100.0)	0.000	0.000	0.999
Mean value of fatigue scale				1.336	0.980-1.821	0.067
Severe fatigue	[≤ 4]	7 (53.8)	6 (46.2)			
	> 4	18 (25.7)	52 (74.3)	3.370	1.021-11.360	0.047
SF-36 physical health score				0.976	0.957-0.994	0.011
SF-36 mental health score				0.978	0.959-0.997	0.022
SF-36 general health score				0.972	0.952-0.992	0.008

[ ] = reference category; SDI = Systemic Lupus International Collaborating Clinics/American College of Rheumatology damage index; OR = odds ratio; CI = confidence interval; SLEDAI = Systemic Lupus Erythematosus Disease Activity Index; anti-dsDNA Ab = anti-double-stranded deoxyribonucleic acid antibodies; sMCP1 = serum monocyte chemoattractant protein-1; uMCP1 = urinary monocyte chemoattractant protein-1; CNS = central nervous system; SF-36 = Medical Outcome Survey Short Form 36; BMD = bone mineral density.

Table 3presents the results from multivariate logistic regression analysis that aimed to assess the impact of multiple factors and to single out those with statistical significance regarding organ damage in SLE patients. The model had nine independent variables, as follows: age, disease duration, physician’s global assessment, use of hydroxychloroquine, use of azathioprine, severe fatigue and SF-36 scores for physical, mental and general health. The whole model was statistically significant (χ^2^ = 40.250; P < 0.001). The model was able to completely account for 39.9% to 57.0% of damage variance. Only five variables made a statistically significant contribution to the model (physician’s global assessment grades 1 and 2, SF-36 physical health, SF-36 mental health and SF-36 general health). Physician’s global assessment grade 2 had the highest odds ratio (OR = 31.839; P = 0.010), followed by physician’s global assessment grade 1 (OR = 16.927; P = 0.012), SF-36 general health (OR=0.901; P = 0.034), SF-36 mental health (OR = 0.899; P = 0.034) and SF-36 physical health (OR = 0.856; P = 0.034).

**Table 3.** Multivariate logistic regression analysis on independent factors among systemic lupus erythematosus patients

Factor		OR	95% CI	P
Age	continuous	1.042	0.944-1.150	0.418
Disease duration	continuous	1.085	0.966-1.218	0.167
Hydroxychloroquine	[no]			
	yes	0.347	0.049-2.446	0.288
Azathioprine	[no]			
	yes	3.256	1.369-5.639	0.921
Physician’s global assessment	[0]			
	1	16.927	1.885-151.961	0.012
	2	31.839	2.327-435.585	0.010
	3	5.405	0.340-86.027	0.232
Severe fatigue	[≤ 4]			
	> 4	0.509	0.050-5.155	0.568
SF-36 physical health score	continuous	0.856	0.752-0.956	0.034
SF-36 mental health score	continuous	0.899	0.899-0.963	0.034
SF-36 general health score	continuous	0.901	0.874-0.985	0.034

OR = odds ratio; CI = confidence interval; SF-36 = medical outcome survey short form 36.

## DISCUSSION

Since organ damage is the principal predictor of mortality among SLE patients,^2^^,^^3^ it is necessary to establish the risk factors for organ damage with permanent sequelae or irreversible loss of function. This formed the aim of the present study. In addition to demographic, clinical and therapeutic factors, the levels of antinucleosome, anti-C1q antibodies and several cytokines like MCP-1, measured in serum and urine, can be estimated as potential risk factors for organ damage in SLE patients. Therehave been several reports on the significance of these biological markers in relation to SLE,^10^^,^^11^^,^^12^^,^^13^ but their impact on the appearance of permanent sequelae and irreversible loss of function has not been reported often.

In this study, the patients with organ damage and SDI ≥ 1 were statistically significantly older than those without organ damage^14^ and had longer disease duration.^15^ In addition, neuropsychiatricand musculoskeletal changes were the most common ones, similar to what was reported from a study conducted in Portugal.^16^ The percentage of cardiovascular events in the present study was within the range of values reported from a meta-analysis on the predictors of cardiovascular events among patients with SLE.^17^

The SDI correlated positively with patient age, disease duration, disease activity and severity of fatigue but negatively with the summary domains of the SF-36 survey: physical, mental and general health. The data indicated that SLE patients with higher damage index had worse quality of life and greater severity of fatigue. The results obtained so far have been divergent regarding the correlation between the degree of damage and quality of life.^5^^,^^6^^,^^18^^,^^19^^,^^20^

In contrast to the disease activity index, the SDI did not show correlations with anti-dsDNA, antinucleosome or anti-C1q antibodies, or with sMCP-1 and uMCP-1. Although serum and urine MCP-1 are good markers of disease activity, they were not shown here to be indicators of organ damage, and neither were antibody levels.^21^^,^^22^

The results from univariate analyses in the present study showed that age, longer disease duration (especially when it was over 10years), higher physician’s global assessment scores, use of azathioprine and greater severity of fatigue were independent risk factors for organ damage, while use of hydroxychloroquine and better quality of life were protective against organ damage. The results from earlier studies have shown that the risk factors for organ damage are numerous and diverse, including not only demographic factors (male gender, advanced age, longer disease duration and African-Caribbean and Indo-Asian origin) but also increased disease activity and renal and CNS involvement.^15^

The time elapsed from the onset of symptoms to diagnosis, lower education levels and presence of a large number of criteria at diagnosis did not show statistical significance in the present study. In a large international study by Bruce et al.,^3^ the socioeconomic status factor (represented as education) was not significantly associated with organ damage.

It is very interesting to examine the use of cytostatic agents, corticosteroids and antimalarial agents for treating SLE, since their use can possibly be correlated with organ damage. Over recent years, increasing amounts of data on organ damage caused through use of corticosteroids and their cumulative dose have become available. Thus, it has been recommended that doses of corticosteroids should be as low as possible.^1^^,^^3^^,^^16^^,^^19^^,^^22^^,^^23^ In the present study, use of corticosteroids was not shown to be a risk factor, as also seen in some other studies.^24^ The same was seen in relation to mycophenolate mofetil and pulse doses of cyclophosphamide. Use of azathioprine was an independent risk factor, similar to what was reported by Sutton et al.^15^ The favorable effects of hydroxychloroquine regarding organ damage prevention in cases of SLE that was obtained in the present study were shown to be similar to those observed in other investigations.^25^^,^^26^

Hypertension, coronary disease, stroke, presence of peripheral atherosclerosis, proteinuria, renal involvement, CNS involvement, osteopenia, or osteoporosis, could not be singled out as independent risk factors for cumulative organ damage, according to the results from univariate analysis in the present study.

The presence of antiphospholipid antibodies can predict the development of neuropsychiatric damage.^27^ In this light, and based on the situation that 13 out of the 83 SLE cases in the present study presented antiphospholipid syndrome, the reason why no significant association was shown here was probably because of the smaller number of respondents or the therapy applied or the shorter duration of monitoring, in comparison with other investigations.

Quality of life, as a separate category, was analyzed in some studies, bearing in mind that SLE is a chronic disease.^28^^,^^29^ In the present study, quality of life was assessed using the standardized Medical Outcome Survey Short Form 36*.* The results from univariate and multivariate analysis showed that better quality of life, especially within the physical and mental domains, was associated with a lower degree of organ damage. The main determinant of organ damage, according to some studies, is overall disease activity.^30^^,^^31^^,^^32^ The multivariate analysis in the present study showed that disease activity evaluated on the basis of physician’s global assessment was the most important risk factor for organ damage.

The strength of the present study is the fact that many parameters were taken into consideration as potential risk factors for organ damage in SLE patients: demographic, clinical, therapeutic and serological. Most other studies did notconsider such a wide range of parameters. One limitation of our study was the number of participants. Future prospective study with larger numbers of participants, in which demographic, clinical, therapeutic and serological parameters are monitored, should provide stronger evidence of risk factors for organ damage in SLE patients.

## CONCLUSION

Although this study was based on a relatively small number of respondents, it showed that the risk factors for organ damage were age, disease duration (especially when this was over 10 years), disease activity, use of azathioprine and severe fatigue. The factors affording protection against organ damage were use of hydroxychloroquine and better quality of life, as expressed through physical, mental and general health scores in a SF-36 survey. Physician’sglobal assessment has a prominent place in the evaluation of disease activity, since it is a simple method and is the most important predictor of organ damage in everyday clinical practice.
